# Predicting Risk of Stroke From Lab Tests Using Machine Learning Algorithms: Development and Evaluation of Prediction Models

**DOI:** 10.2196/23440

**Published:** 2021-12-02

**Authors:** Eman M Alanazi, Aalaa Abdou, Jake Luo

**Affiliations:** 1 Department of Health Informatics College of Health Sciences Saudi Electronic University Riyadh Saudi Arabia; 2 Department of Biomedical and Health Informatics College of Engineering University of Wisconsin-Milwaukee Milwaukee, WI United States; 3 Department of Radiotherapy Children's Cancer Hospital 57357 Cairo Egypt; 4 Department of Health Informatics and Administration College of Health Sciences University of Wisconsin-Milwaukee Milwaukee, WI United States

**Keywords:** stroke, lab tests, machine learning technology, predictive analytics

## Abstract

**Background:**

Stroke, a cerebrovascular disease, is one of the major causes of death. It causes significant health and financial burdens for both patients and health care systems. One of the important risk factors for stroke is health-related behavior, which is becoming an increasingly important focus of prevention. Many machine learning models have been built to predict the risk of stroke or to automatically diagnose stroke, using predictors such as lifestyle factors or radiological imaging. However, there have been no models built using data from lab tests.

**Objective:**

The aim of this study was to apply computational methods using machine learning techniques to predict stroke from lab test data.

**Methods:**

We used the National Health and Nutrition Examination Survey data sets with three different data selection methods (ie, without data resampling, with data imputation, and with data resampling) to develop predictive models. We used four machine learning classifiers and six performance measures to evaluate the performance of the models.

**Results:**

We found that accurate and sensitive machine learning models can be created to predict stroke from lab test data. Our results show that the data resampling approach performed the best compared to the other two data selection techniques. Prediction with the random forest algorithm, which was the best algorithm tested, achieved an accuracy, sensitivity, specificity, positive predictive value, negative predictive value, and area under the curve of 0.96, 0.97, 0.96, 0.75, 0.99, and 0.97, respectively, when all of the attributes were used.

**Conclusions:**

The predictive model, built using data from lab tests, was easy to use and had high accuracy. In future studies, we aim to use data that reflect different types of stroke and to explore the data to build a prediction model for each type.

## Introduction

Stroke is a neurological deficit, primarily because of acute central nervous system focal injury caused by a vascular issue. It is a major cause of disability and death worldwide [1]. It estimated that the overall prevalence of stroke in the United States is 2.5%, and about 7 million Americans over the age of 20 years have experienced a stroke. The condition has a significant negative impact on patients’ health and quality of life. It also has a negative impact on hospital services and the availability of beds and was estimated to cost the US economy about US $351.2 billion between 2014 and 2015 [2]. There are two types of stroke: ischemic and hemorrhagic. Hemorrhagic stroke occurs because of a burst vessel that leads to bleeding in the brain, whereas ischemic stroke occurs because of a blockage of the arteries of the brain. Ischemic strokes are the most common, comprising 85% to 90% of all strokes [3]. This condition can be prevented by promoting health and increasing awareness of risk factors. There are many risk factors related to lifestyle, including obesity, diet, alcohol intake, and lack of physical activity [4]. Underlying conditions, such as diabetes, hypertension, and cardiovascular diseases, may also lead to stroke. Therefore, proper self-management of these diseases and the pursuit of a healthy lifestyle may prevent the occurrence of stroke.

In 2019, the American College of Cardiology/American Heart Association released the Guideline on the Primary Prevention of Cardiovascular Disease. The guideline recommends a complete assessment and examination of patients who are at risk of developing blockages in their arteries that may lead to a heart attack or stroke and might die as a result [5]. Now more than ever, physicians can access clinical evidence to identify high-risk patients using approaches such as acquiring a complete patient history and conducting thorough physical exams for risk assessment. Patient records contain many useful predictive factors, such as patient demographic (eg, age and gender), lifestyle (eg, diet and physical activity), and existing medical condition factors (eg, diabetes and hypertension), that might lead to stroke [5]. The growth of arterial blockages and decades of damage to blood vessels, which may lead to stroke, are often associated with these risk factors. If physicians can assess the risks of stroke easily and conveniently, strokes could be prevented at an earlier stage. This approach could save lives and reduce the economic burden of health care services. In the age of artificial intelligence and machine learning, a clinical decision support system has been developed to assist physicians to diagnose and identify individuals with a high risk of stroke. The potential of applying machine learning technologies in the cardiovascular domain is significant, from identifying individuals with a high risk of stroke [6,7] to predicting outcomes of patients following treatment [8,9]. Most of these studies use either health habits and lifestyle factors, such as smoking or alcohol consumption; conditions that predispose to strokes, such as hypertension and diabetes mellites; or neuroimaging, such as computed tomography and magnetic resonance imaging, to either classify or predict the disease.

Besides assessing known risk factors for stroke, scientists are trying to develop lab tests that can predict stroke. One of the major advantages of using lab test results for prediction is that lab tests are commonly collected in clinical settings, and the information is often well documented in patients’ records. In this study, we explored data-driven approaches using supervised machine learning models to predict the risk of stroke from different lab tests.

Several studies have been able to identify independent laboratory tests that are correlated with stroke using descriptive statistical analysis. Sughrue et al [10] conducted a retrospective study in 2013 that identified 35 tests with a statistically significant correlation with a future stroke diagnosis. The most informative were for various types of cholesterol. Two of these 35 laboratory tests were urine tests, and 33 were blood, serum, or plasma tests. Some tests were positively associated with an outcome of stroke (ie, neutrophil count and percent; CD3+, CD8+, and T8 suppressor cells; monocytes; eosinophils; and CD3 cells), and others were negatively correlated (ie, hematocrit and lymphocytes). Their results show that it is possible to correlate future stroke with collected lab test data. Farah and Samra [11] conducted a retrospective study investigating the association between the neutrophil-to-lymphocyte ratio (NLR), mean platelet volume (MPV), and the risk of stroke. Two-tailed *t* tests showed no significant differences in the stroke group’s MPV values compared with those in the control group. However, the NLRs of the stroke patients were significantly different compared with those of the control group. That study indicated the existence of a correlation between the level of NLR and stroke risk. NLR levels have been shown to be higher in stroke patients than in control groups. Feng et al [12] reviewed the scientific literature on the potential role and the possible epidemiological relationships between red cell distribution width (RDW) and ischemic stroke in a meta-analysis of 40 manuscripts from China National Knowledge Infrastructure and PubMed databases. They reported that patients with stroke had higher levels of RDW than those without strokes. Another study by Kaya et al [13] also investigated the association between baseline RDW level and stroke risk in patients with heart failure. These authors found that heart failure patients suffering from stroke had significantly increased basal RDW levels (mean 16.9, SD 1.14, vs mean 14.8, SD 1.6; *P*<.001) and serum uric acid levels (mean 8.8, SD 1.7, vs mean 7.5, SD 1.1; *P*=.027) compared with patients without stroke, according to the propensity score analysis. Giles et al [14] used data from a national cohort to investigate whether low folate levels were associated with ischemic stroke and found that folate concentrations of ≤9.2 nmol/L could be a risk factor for ischemic stroke (relative risk 1.37, 95% CI 0.82-2.29). Another study by Qin et al [15] concluded that there is a significant risk of first ischemic stroke in hypertensive patients with low levels of folate and vitamin B12.

These studies demonstrate the value of lab test results for predicting stroke. Our study aimed to leverage lab test results to build machine learning models for stroke prediction. We prepared the data sets using three data selection techniques for this study. After that, for each data selection technique, we applied four individual machine learning classifiers to prepare prediction models. We measured the performance of each prediction model using six different performance measures. Our results indicate that the data resampling technique outperformed the decision tree and random forest classifiers.

## Methods

### Overview

**Figure 1:**
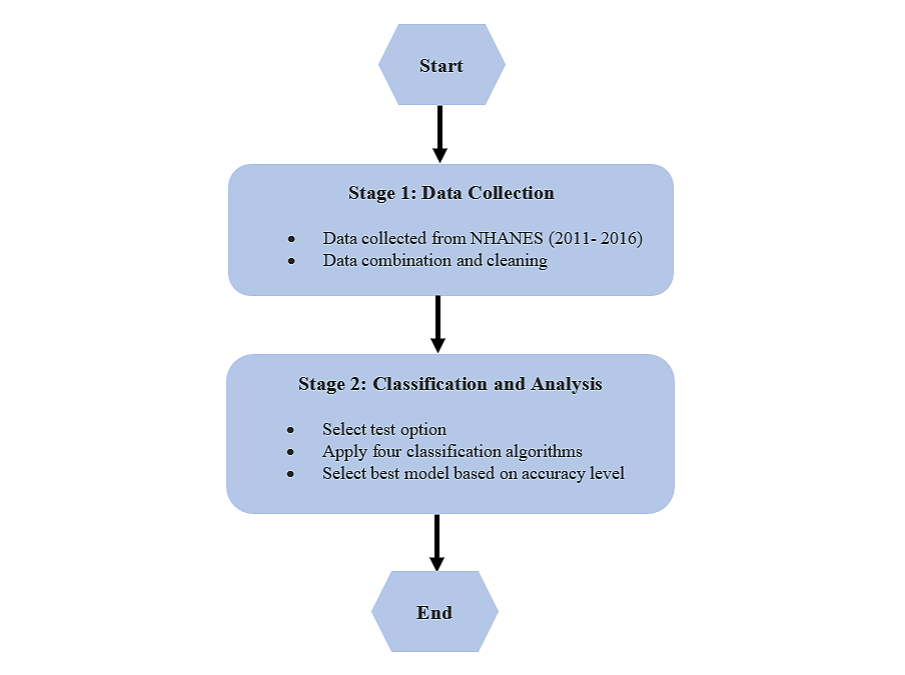
Flow diagram of the study methodology. NHANES: National Health and Nutrition Examination Survey.

Figure 1 shows the outline of our investigation. In the first step, we collected data from the National Health and Nutrition Examination Survey (NHANES). In the second step, we selected the data using three data techniques for our prediction models. The first one was conducted without data resampling, the second one included data imputation, and the third one was conducted with data resampling.

We used 10-fold cross-validation to perform the train and test approach. To train models, we used four different machine learning classifiers, and six performance measures were used to assess the performance of the models. The elaborated descriptions of the data sets, classifiers, and performance metrics that were used are given below.

### Data Collection

**Figure 2:**
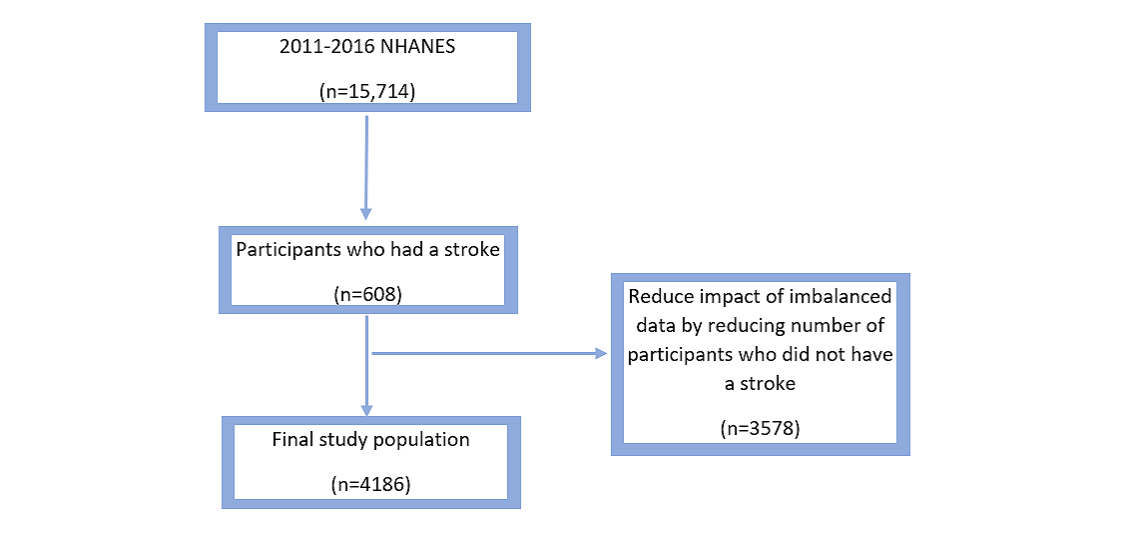
Participant selection and prevalence of stroke in the National Health and Nutrition Examination Survey (NHANES).

The NHANES survey was conducted to examine the health and nutritional status of adults and children in the United States; “NHANES is a major program of the National Center for Health Statistics (NCHS). NCHS is part of the Centers for Disease Control and Prevention (CDC) and has the responsibility for producing vital and health statistics for the Nation” [16]. The data sets contain five domains: demographics, dietary data, examination data, laboratory data, and questioner data. Each domain contains several subdomains. Our focus was on data sets that contain information about laboratory tests. The data sets are available from 1999 to 2017, and we considered data from 2011 to 2015. The total number of participants was 15,714 during this period. To reduce the impact of imbalanced data, we noted that in the entire data set, there were about 17% of participants who had experienced a stroke. Therefore, we included total of 4186 participants, of whom 608 (14.5%) had experienced a stroke (Figure 2). The list of data attributes is shown in Table 1. The data sets contained 21 attributes, including each patient’s age and gender as well as other lab test information for each respective patient. The data sets and their information are available online [16], where the data are presented from the year 2000 to the current year. For this study, the data were collected for each year and combined using the sequence number (SEQN). After combining and cleaning the data, we used the Waikato Environment for Knowledge Analysis (WEKA; version 3.8.0) system to build and test machine learning models.

**Table 1:** List of the data attributes.

Feature^a^	Units
Age	Years
Gender	N/A^b^
Albumin, urine	ug/mL
Creatinine, urine	mg/dL
White blood cell count	1000 cells/μL
Lymphocytes	1000 cells/μL
Monocytes	1000 cells/μL
Segmented neutrophils	1000 cells/μL
Eosinophils	1000 cells/μL
Basophils	1000 cells/μL
Red blood cell count	Million cells/μL
Hemoglobin	g/dL
Hematocrit	%
Mean cell volume	fL
Mean cell hemoglobin	pg
Mean corpuscular hemoglobin concentration	g/dL
Red cell distribution width	%
Platelet count	1000 cells/μL
Mean platelet volume	fL
Cotinine, serum	ng/mL
Red blood cell folate	mg/dL

^a^All data types were numeric, except for “gender,” which was nominal.

^b^N/A: not applicable; this type of data did not have units.

### Classification

Several different machine learning algorithms can handle a binary classification problem. In this study, we used four machine learning algorithms: naïve Bayes, BayesNet, J48 (Java implementation of C4.5 algorithm), and random forest. The performance of the algorithms was evaluated and compared for stroke prediction using lab test results as features. Details of the algorithms are as follows:

- The J48 algorithm creates a tree based on the C4.5 algorithm with pruning.
- The random forest algorithm creates a forest of random trees and outputs the mode of the classes created by individual trees.
- The naïve Bayes algorithm creates a classifier based on the naïve Bayes method, which assumes that all attributes are independent.
- The BayesNet algorithm creates a classifier based on non–naïve Bayes, which does not assume that all attributes are independent.

In the cross-validation approach, the data sets are divided into several equal portions; in general, 5-fold and 10-fold cross-validations are used when the data sets are equally divided into 5 and 10 portions [17]. With this approach, for each simulation, one portion of each data set is used to train the prediction model and the rest are used for validation. In this study, we used 10-fold cross-validation and, in this process, we divided the whole of each data set into 10 equal parts; each time, 10% of each data set was used to train the model and 90% was used for validation. In this task, three data analyses were conducted where the first data analysis applied each of the machine learning techniques on the data sets without data manipulation or resampling. The aim was to determine the baseline for the data sets among the various machine learning techniques. The imputation of missing data set entries was conducted in the second analysis. In statistics, imputation entails substituting missing data with values calculated using any of a number of techniques [18]. Imputation is a useful technique in remedying missing data, since missing data may lead to inaccurate predictions. We used the default ReplaceMissingValue filter in WEKA, which replaces all missing values for nominal and numeric attributes in a data set with the modes and means from the training data. Most of the features had 5% missing values, and one feature had 11% missing values. After the imputation of the missing data, data resampling was conducted in the third analysis. Data resampling is a commonly used technique, since training may result in nonuniformity of class labels. In this case, the resampling technique was applied to select a specific subset of data points for model training [19]. After resampling the data, the results of the first analysis should be improved because of the balancing of the data set distribution. A balanced distribution was achieved through the use of WEKA, which randomly resamples the data. Based on the available theoretical knowledge about resampling and imputation in statistics, the results after the third analysis should be improved.

### Evaluation Metrics

Model accuracy was evaluated based on the following measures: recall or sensitivity, specificity, positive predictive value (PPV), negative predictive value (NPV), accuracy, and area under the curve (AUC) (or area under the receiver operating characteristic [ROC] curve) to compare the four classifiers. Details of these measures are as follows:

- Sensitivity, also known as recall or true positive rate, is the number of true positives divided by the number of true positives plus the number of false negatives. It is the likelihood that the patient has a high risk of stroke [20].
- Specificity, also known as the true negative rate, is the proportion of individuals classified as nonstroke to the total number of actual nonstroke cases. It is the likelihood that a patient who does not have a risk of stroke will have a negative result [21].
- PPV, also known as precision, is the number of true positives divided by the number of true positives plus the number of false positives. It is the proportion of individuals who have suffered a stroke to the total number of participants classified as having a risk of stroke [22].
- NPV is the percentage of negative tests in patients who are free from the disease or the proportion of individuals who have not suffered a stroke to the total number of participants classified as not having a risk of stroke [22].
- Overall accuracy is the number of correctly classified instances over the total size of the data set [20].
- The AUC is the area under the ROC curve, which is constructed by plotting the true positive rate against the true negative rate [23].

We will also look at the Pearson correlation coefficient value of each independent predictor to investigate the relationship between each lab test and risk of stroke.

## Results

In the NHANES data sets, 608 participants suffered from a stroke from 2011 to 2015. The median age of participants who had a stroke was 51 years for both men and women. The numbers of men and women who had a stroke were 220 (36.2%) and 190 (31.3%), respectively; 198 (32.6%) participants did not reveal their gender identity.

After the data collection process, the data were analyzed in three ways: without data resampling, with data imputation, and with data resampling. Data resampling techniques were used to tackle data imbalance problems in the data sets. These sampling techniques are widely used in machine learning–based prediction models in different areas [24]. Our first analysis was done without the data resampling technique, where the four machine learning algorithms were applied directly to the data sets. The first analysis produced poor results for all four classifiers. The best sensitivity rate among the classifiers in the first analysis was for the BayesNet model, followed by the naïve Bayes model. In the second analysis, we applied the data imputation technique to the data sets, which replaced missing values and deleted features that had more than 50% missing values; the prediction accuracy improved for all models, except for the naïve Bayes model, whose performance decreased slightly after replacing the missing values.

**Figure 3:**
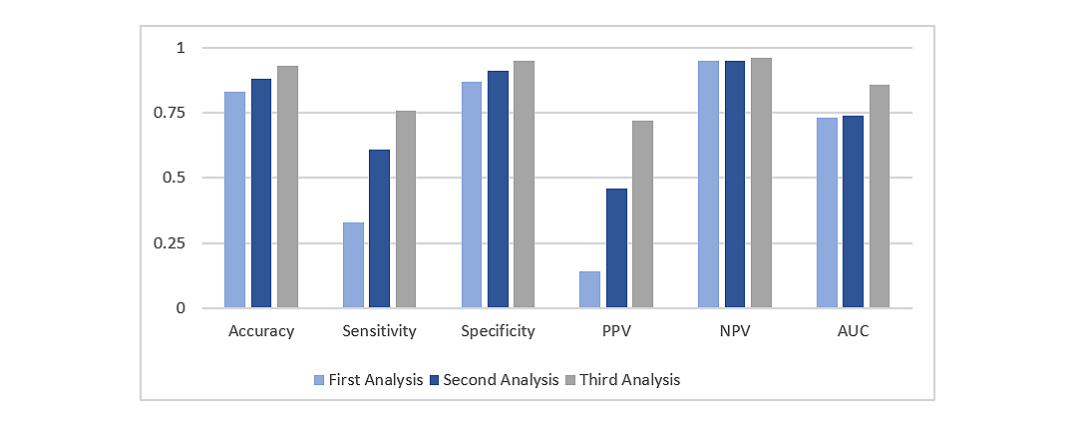
Performance comparison among three data selection techniques for the decision tree model. AUC: area under the curve; NPV: negative predictive value; PPV: positive predictive value.

**Figure 4:**
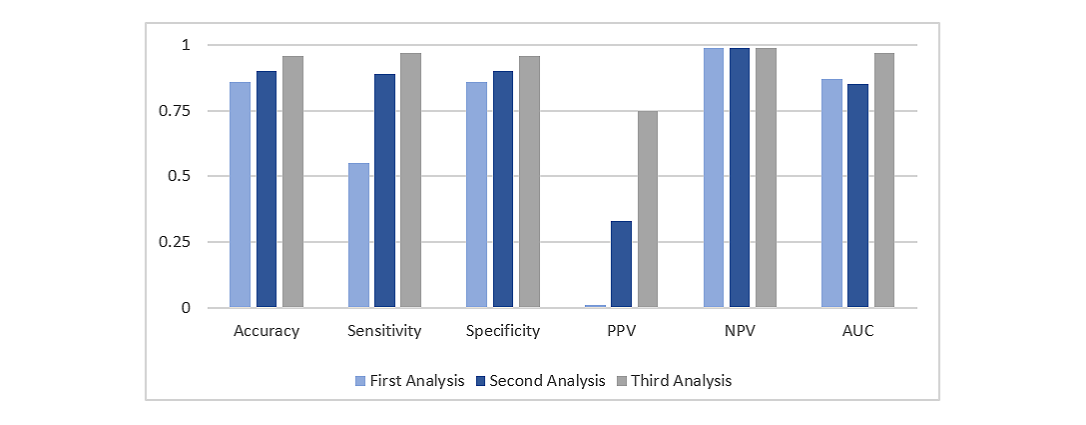
Performance comparison among three data selection techniques for the random forest model. AUC: area under the curve; NPV: negative predictive value; PPV: positive predictive value.

In the third analysis, we resampled the data. After resampling, the prediction accuracy improved significantly for both the decision tree and random forest models, but only slightly for the naïve Bayes and BayesNet models. Table 2 shows the scores of accuracy, sensitivity, specificity, PPV, NPV, and AUC, according to the three data analysis techniques and four classifiers. The table shows that the random forest model was the best classifier with the data resampling technique. Figures 3 and 4 show the score comparisons among the three data selection techniques for the decision tree and random forest models, respectively. We considered the decision tree and random forest classifiers to compare the performance, as they significantly improved the performance in the third analysis. Both figures clearly show that the third analysis, the data resampling technique, outperformed the other two techniques for the decision tree and random forest classifiers.

**Table 2:** Results of three data analysis techniques.

Technique and classifier		Accuracy	Sensitivity	Specificity	PPV^a^	NPV^b^		AUC^c^	
**Without data resampling**									
	Naïve Bayes	0.82	0.34	0.88	0.27		0.91		0.76
	BayesNet	0.82	0.38	0.89	0.37		0.90		0.88
	Decision tree	0.83	0.33	0.87	0.14		0.95		0.73
	Random forest	0.86	0.55	0.86	0.01		0.99		0.87
**Data imputation**									
	Naïve Bayes	0.81	0.32	0.88	0.25		0.91		0.74
	BayesNet	0.86	0.53	0.92	0.54		0.92		0.85
	Decision tree	0.88	0.61	0.91	0.46		0.95		0.74
	Random forest	0.90	0.89	0.90	0.33		0.99		0.85
**Data resampling**									
	Naïve Bayes	0.82	0.33	0.88	0.29		0.90		0.74
	BayesNet	0.87	0.53	0.93	0.57		0.92		0.85
	Decision tree	0.93	0.76	0.95	0.72		0.96		0.86
	Random forest	0.96	0.97	0.96	0.75		0.99		0.97

^a^PPV: positive predictive value.

^b^NPV: negative predictive value.

^c^AUC: area under the curve.

Table 3 shows the results from Pearson correlation analysis of the independent predictors.

**Table 3:** Pearson correlation coefficient values of independent predictors.

Independent predictor of stroke	Pearson correlation coefficient (*r*)
Age	0.26
Gender	0.13
Red cell distribution width (%)	0.18
Lymphocytes (%)	0.15
Red blood cell folate (ng/mL)	0.13
Segmented neutrophils (%)	0.12
Hemoglobin (g/dL)	0.11
Red blood cell count (million cells/μL)	0.11
Hematocrit (%)	0.09
Lymphocytes (1000 cells/μL)	0.08
Segmented neutrophils (1000 cell/μL)	0.07

## Discussion

### Principal Findings

From the previous section, we noticed that our models had the potential to perform stroke prediction using lab test data. Our results show that the random forest model was the best classifier after conducting the data resampling technique.

Also, several observations can be made from the results in Table 3. We identified nine lab tests, in addition to age and gender, that effectively correlated with stroke occurrence. These correlations were calculated using the Pearson correlation coefficient. These results align with other research that showed a linear relationship between some of these variables and stroke. Several studies have shown that age is correlated with the risk of stroke. According to Muntner et al [2], stroke incidence doubles after the age of 45 years, and 70% of all strokes occur over the age of 65 years. Many studies have investigated the relationship between baseline RDW and stroke. They found that elevated RDW is a risk factor in ischemic stroke [12,13,25]. One of the novel correlations that were found in this study is the lymphocyte percentage. Lymphocytes are white blood cells, including B cells, T cells, and natural killer cells. Lymphocyte percentage is positively associated with stroke occurrence. There have been no studies suggesting that lymphocyte percentage can be a predictor of stroke, but different studies have examined the use of immune cells as biomarkers to predict stroke outcome [26,27]. There is one study that showed a negative correlation between hematocrit and stroke occurrence [10]. Folate deficiency has various clinical manifestations. Our finding that serum folate level was correlated with the risk of stroke is in line with the finding of Giles et al [14], who found that a serum folate concentration of ≤9.2 nmol/L may slightly increase the risk for ischemic stroke. Other studies have shown that folic acid therapy involving folic acid, vitamin B12, and vitamin B6 reduced the risk of ischemic stroke [15,28]. Neutrophils, which are normally the most abundant circulating white blood cells and respond quickly to infection, also contribute to the main processes causing an ischemic stroke, as they facilitate the development of blood clots. Neutrophils are, therefore, also of considerable importance as targets for treating and preventing ischemic stroke [29]. A study by Sughrue et al [10] produced results similar to ours regarding the positive association between neutrophils and stroke occurrence. Hemoglobin levels can predict the risk of stroke. Observational studies have reported an independent association between red blood cell count, hematocrit, and hemoglobin concentration and the risk of developing stroke [30,31].

The correlations between these different lab tests and stroke were found in several studies. However, this is the first study that used all of these different attributes to build a prediction model using machine learning algorithms. Our results showed that a prediction model can be created using the random forest algorithm and could achieve an accuracy of 0.96.

### Conclusions

Machine learning applications are becoming more widely used in the health care sector. The prediction of stroke using machine learning algorithms has been studied extensively. However, no previous work has explored the prediction of stroke using lab tests. The results of several laboratory tests are correlated with stroke. Building a prediction model that can predict the risk of stroke from lab test data could save lives. In this study, we created a prediction model using the random forest algorithm and achieved a 96% accuracy rate. The model can be integrated with electronic health records to provide a real-time prediction of stroke from lab tests. Because of the nature of the data, we could not predict the type of stroke: hemorrhagic or ischemic. In future studies, we aim to use data that provide information about different types of stroke to build prediction models for each type.

## Abbreviations

AUC: area under the curve
CDC: Centers for Disease Control and Prevention
MPV: mean platelet volume
NCHS: National Center for Health Statistics
NHANES: National Health and Nutrition Examination Survey
NLR: neutrophil-to-lymphocyte ratio
NPV: negative predictive value
PPV: positive predictive value
RDW: red cell distribution width
ROC: receiver operating characteristic
SEQN: sequence number
WEKA: Waikato Environment for Knowledge Analysis
